# Unraveling Inflammatory Pain: A Narrative Review of Molecular Pathways and Multidisciplinary Treatments

**DOI:** 10.1002/hsr2.72559

**Published:** 2026-05-25

**Authors:** Fatma Mohamed Elmansy, Mohamed Goda Elbqry, Saddam Ahmed Al‐Ahdal, Fatima S. O. Ashmieg, Patience Osose Nasir, Nasiru Mohammed Abdullahi, Mohammed Nader Shalaby

**Affiliations:** ^1^ Department of Medical‐Surgical Nursing, College of Nursing Qassim University Buraydah Saudi Arabia; ^2^ Department of Medical‐Surgical Nursing, Faculty of Nursing Suez Canal University Ismailia City Egypt; ^3^ Department of Medical‐Surgical Nursing, College of Nursing Sciences Inaya Medical Colleges Riyadh Saudi Arabia; ^4^ Department of Community, Psychiatric and Mental Health Nursing, College of Nursing Qassim University Buraydah Saudi Arabia; ^5^ Biological Sciences and Sports Health Department, Faculty of Physical Education Suez Canal University Egypt

**Keywords:** inflammation, molecules, multidisciplinary, pain

## Abstract

**Background:**

Inflammatory pain is a widespread global health concern arising from complex molecular pathways triggered by a consequence of tissue damage and immune activation. It significantly impacts quality of life and functional outcomes, necessitating a comprehensive understanding of underlying mechanisms and multidisciplinary approaches for effective management.

**Aim:**

This review aims to provide an integrative synthesis of the molecular mechanisms underlying inflammatory pain and to critically examine contemporary multidisciplinary therapeutic approaches for its management. It also highlights the essential role of nursing within the framework of precision and presence‐based care.

**Methods:**

A narrative review was conducted using electronic searches across major databases, including SCOPUS, Web of Science, CINAHL, IBSS, and PubMed, to identify relevant English‐language publications from 2020 to 2025. Additional manual searches were performed by screening relevant journals and the reference lists of selected studies. The search strategy incorporated combinations of keywords such as molecular mechanisms of pain, inflammatory pain, nursing, and nurse.

**Results:**

Recent advances in molecular science highlight the critical role of inflammatory mediators and signaling pathways in the development and maintenance of inflammatory pain. Effective management requires multidisciplinary strategies integrating pharmacological interventions, including nonsteroidal anti‐inflammatory drugs (NSAIDs), corticosteroids, and biologic agents, with adjunctive approaches such as physiotherapy, electrostimulation, and psychosocial interventions. Within this framework, nurses play a pivotal role in translating molecular insights into clinical practice by delivering evidence‐based, patient‐centered, and holistic care.

**Conclusion:**

Integrating molecular understanding with multidisciplinary clinical approaches enhances pain assessment accuracy, treatment effectiveness, and patient outcomes. Strengthening nursing competencies in molecular pain mechanisms is essential to support precision care and optimize the management of inflammatory pain.

## 1 Introduction

**Figure 1:**
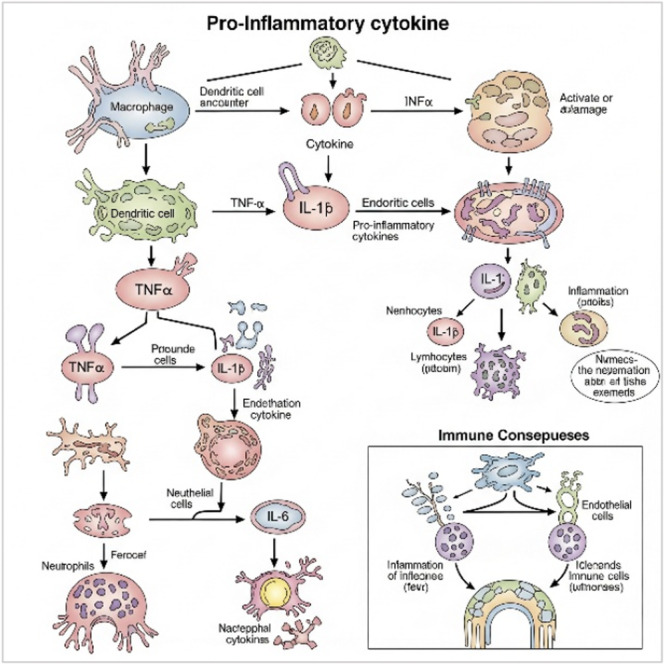
Pro‐inflammatory cytokines and consequences of the immune system.

Inflammatory pain is a highly prevalent and devastating condition that arises in response to tissue injury, infection, or autoimmune processes [1]. It is characterized by hyperalgesia and allodynia, driven by complex interactions between the immune and nervous systems [2]. Unlike nociceptive pain, which is classically acute and resolves with tissue healing, inflammatory pain may persist and transition into a chronic state due to sustained immune activation and neural sensitization [3]. At the molecular level, inflammatory pain is mediated through a cascade of biochemical signals (Figure 1) involving pro‐inflammatory cytokines, prostaglandins, neuropeptides, and ion channels. The process begins when pathogen‐associated molecular patterns (PAMPs) from bacteria are detected by Toll‐like receptors (TLRs) on the surface of immune cells [4]. These mediators contribute to both peripheral sensitizations, where nociceptors in damaged tissue become hyper‐responsive, and central sensitization, where increased excitability occurs in spinal and supraspinal neurons [5]. Recent advances in pain neurobiology have deepened our understanding of these signaling pathways and their modulation using various approaches, including pharmacological agents (e.g., corticosteroids) and integrative interventions (e.g., acupuncture) [6]. Given the multifactorial nature of inflammatory pain, a multidisciplinary approach that combines medical, psychological, and rehabilitative care has emerged as the gold standard in pain management [7].

This figure shows key pro‐inflammatory cytokines (TNF‐α, IL‐1β, IL‐6) released by immune cells in response to injury or infection. These cytokines activate inflammatory pathways (e.g., NF‐κB, MAPK), leading to the production of additional mediators, nociceptor sensitization, and increased pain perception. The figure also highlights immune cell recruitment, tissue damage, and the progression to chronic inflammation, illustrating the overall impact of cytokine‐driven immune responses.

Nurses play an increasingly critical role. Nursing care in pain management has evolved from a primarily supportive function to a highly skilled, knowledge‐driven practice [8]. Nurses are responsible not only for pain assessment and medication administration but also for monitoring therapeutic response, educating patients, and applying non‐pharmacologic pain relief techniques [9]. The increasing focus on precision nursing guided by molecular understanding and individualized patient characteristics has strengthened nurses' capacity to implement tailored, evidence‐based interventions. Accordingly, this review seeks to elucidate the molecular underpinnings of inflammatory pain, evaluate contemporary multidisciplinary therapeutic approaches, and underscore the critical role of nurses in integrating biomedical science with empathetic, presence‐centered care [10].

### 1.1 Aim

The aim of this review is to provide an integrative synthesis of the molecular mechanisms underlying inflammatory pain and to critically examine contemporary multidisciplinary therapeutic approaches for its management, including pharmacological, behavioral, physical, and interventional strategies. Within this broader multidisciplinary framework, the review also highlights the essential role of nursing in delivering precision and presence‐based care, emphasizing how nurses contribute to comprehensive, patient‐centered pain management through evidence‐informed practice, coordination of care, and holistic support.

## 2 Methods

### 2.1 Study Design

A narrative review design was employed to provide a comprehensive synthesis of the existing literature on inflammatory pain, including its molecular mechanisms and multidisciplinary management approaches.

### 2.2 Search Strategy

An extensive literature search was conducted using major electronic databases, including SCOPUS, Web of Science (WOS), CINAHL, IBSS, and PubMed. To ensure comprehensive coverage, manual searches were also performed by screening relevant journals and the reference lists of key publications. The search was limited to studies published in English between 2020 and 2025. A combination of keywords was used, including “molecular mechanisms of pain,” “inflammatory pain,” “nursing,” and “nurse.”

### 2.3 Inclusion Criteria

Studies were included if they were peer‐reviewed articles, systematic reviews, narrative reviews, or clinical guidelines published in English between 2020 and 2025. Eligible studies addressed the mechanistic or therapeutic aspects of inflammatory pain, with particular emphasis on molecular or biochemical pathways and the role of nursing in pain management, particularly within the frameworks of precision nursing and presence‐based care.

### 2.4 Exclusion Criteria

Studies were excluded if they focused exclusively on neuropathic, cancer‐related, or postoperative pain without an inflammatory component. Additionally, non–peer‐reviewed publications, such as opinion papers, editorials, or commentaries lacking empirical evidence, were excluded. Non‐English studies and those lacking clinical or multidisciplinary relevance to nursing practice were also excluded. Furthermore, studies limited to pediatric or animal models without clear implications for adult human care were not considered.

### 2.5 Data Extraction and Synthesis

The review process involved identifying the research problem, systematically screening and selecting relevant studies, and extracting key information related to molecular mechanisms, therapeutic approaches, and nursing roles in inflammatory pain management. The selected studies were critically analyzed and narratively synthesized to provide an integrated understanding of the topic and to highlight current evidence, gaps, and implications for clinical practice.

## 3 An Overview of Molecular Mechanisms and Multidisciplinary Therapeutic Strategies of Inflammatory Pain

### 3.1 Inflammatory Pain

**Figure 2:**
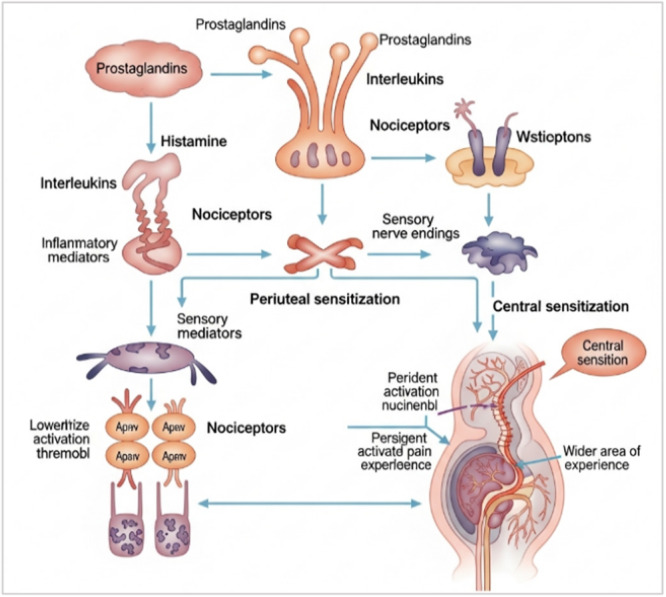
Inflammatory pain.

Inflammatory pain represents a subtype of nociceptive pain (Figure 2) that arises from immune‐mediated responses following tissue injury, infection, or autoimmune dysregulation [11]. This process is characterized by the release of inflammatory mediators, which sensitize peripheral nociceptors and increase the excitability of central neurons. While acute inflammatory pain serves a protective physiological function, facilitating tissue repair and promoting rest, sustained inflammation may induce maladaptive neuroplastic changes such as central sensitization, ultimately contributing to chronic pain states [12].

Globally, inflammatory pain imposes a substantial health burden, with an estimated 20% of adults experiencing chronic pain of inflammatory origin [13]. Among these conditions, rheumatoid arthritis (RA) is one of the most extensively studied, affecting approximately 0.5%–1% of the world's population, with a higher incidence among women and older adults [14]. Similarly, osteoarthritis (OA) traditionally viewed as a degenerative joint disease also encompasses a significant inflammatory component, impacting over 300 million individuals worldwide. Moreover, inflammatory pain is a hallmark of several chronic inflammatory disorders, including inflammatory bowel disease (IBD), further highlighting its pervasive impact on global health [15].

This figure illustrates the mechanisms underlying inflammatory pain, beginning with the release of inflammatory mediators such as prostaglandins, interleukins, and histamine from damaged tissues and immune cells. These mediators activate and sensitize nociceptors (pain receptors), lowering their activation threshold and enhancing pain signaling (peripheral sensitization). The signals are then transmitted through sensory nerve endings to the central nervous system, where persistent stimulation leads to central sensitization, characterized by amplified pain perception and an expanded area of pain. The figure also highlights the role of ion channels and ongoing nociceptor activation in maintaining prolonged pain responses.

**Figure 3:**
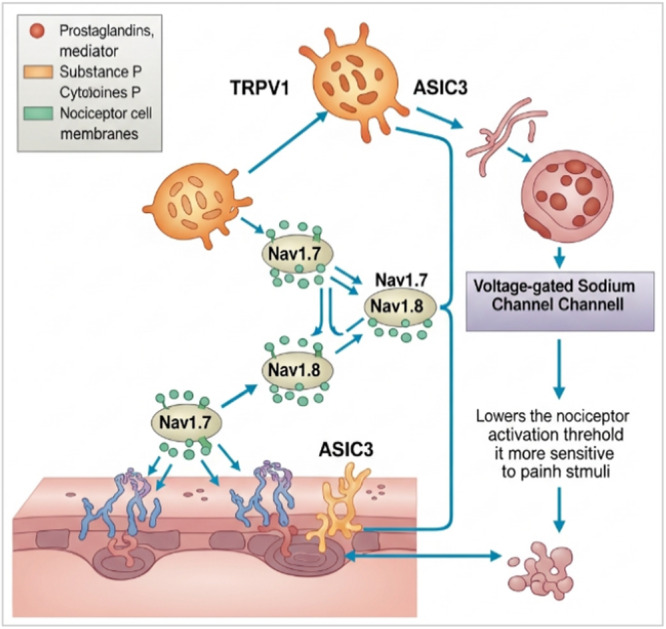
Peripheral inflammatory pain.

The pathophysiology of inflammatory pain involves both peripheral and central processes. In the periphery (Figure 3), nociceptors are sensitized by inflammatory mediators through the activation of ion channels (e.g., TRPV1 and ASIC3) and voltage‐gated sodium channels (e.g., Nav1.7 and Nav1.8), leading to a reduced threshold for activation [16]. Persistent nociceptive input to the spinal cord results in increased synaptic transmission, activation of glial cells, and heightened excitability of dorsal horn neurons, a phenomenon known as central sensitization [17]. Certain populations are at higher risk of developing inflammatory pain, including older adults due to age‐related immune changes and degenerative diseases, women (particularly because of hormonal and genetic factors), individuals with a family history of autoimmune diseases, and patients undergoing major surgeries or those with chronic infections [18]. Clinically, patients often present with localized or generalized pain, swelling, morning stiffness, fatigue and, in some cases, systemic symptoms such as fever or malaise, depending on the underlying cause [19].

This figure illustrates the molecular mechanisms of peripheral inflammatory pain, focusing on the role of ion channels and inflammatory mediators at the nociceptor level. Inflammatory mediators such as prostaglandins, substance P, and cytokines interact with nociceptor cell membranes, leading to the activation and modulation of key ion channels, including transient receptor potential vanilloid 1 (TRPV1), acid‐sensing ion channels (ASIC3), and voltage‐gated sodium channels (Nav1.7 and Nav1.8). These channels enhance neuronal excitability by facilitating sodium influx and depolarization of nociceptive neurons. As a result, the activation threshold of nociceptors is lowered, making them more sensitive to painful stimuli. This process contributes to peripheral sensitization and the amplification of pain signals at the site of inflammation.

The diagnosis of inflammatory pain involves a thorough assessment, including clinical history, physical examination, laboratory tests (e.g., erythrocyte sedimentation rate), and imaging techniques (e.g., MRI) to identify joint or soft tissue inflammation [20]. Additionally, emerging biomarkers, including cytokine profiles, are being investigated to enhance diagnostic accuracy [21]. Unmanaged inflammatory pain can result in significant complications. Chronic inflammation may cause joint, central sensitization with widespread pain, reduced mobility, psychological issues, and a general decline in quality of life [22]. Consequently, early and precise diagnosis, along with a comprehensive, multidisciplinary treatment approach, is essential to prevent long‐term disability and improve patient outcomes [23].

### 3.2 Molecular Mechanisms of Inflammatory Pain

Inflammatory pain results from the intricate interactions between the immune system and nervous system responses. It is commonly triggered by tissue injury, infections, or autoimmune conditions that activate inflammatory signaling cascades [24]. These cascades lead to the release of diverse molecular mediators that engage with nociceptors specialized sensory neurons that detect potentially damaging stimuli. This interaction promotes both peripheral and central sensitization, key mechanisms in the persistence and amplification of pain [22]. This review examines the molecular underpinnings of inflammatory pain, highlighting the roles of inflammatory mediators, ion channels, neuropeptides, and central nervous system sensitization in its pathophysiology [25].

#### 3.2.1 Inflammatory Mediators in Peripheral Sensitization

Peripheral sensitization occurs when tissue injury causes the release of inflammatory mediators or immune activation lowers the activation threshold of nociceptors, making them more responsive to pathophysiological stimuli [26]. Key mediators include prostaglandin E2 (PGE2), bradykinin, tumor necrosis factor‐alpha, and interleukins such as IL‐1β and IL‐6. These substances activate intracellular signaling pathways in primary afferent neurons, leading to increased expression and activity of ion channels and receptors involved in pain transmission (e.g., TRPV1, Nav1.8) pathophysiology [27]. This results in heightened pain sensitivity (hyperalgesia) and pain in response to normally non‐painful stimuli (allodynia). Persistent release of these mediators contributes to the transition from acute to chronic pain [28].

#### 3.2.2 Ion Channel Modulation in Nociceptors

Nociceptors detect inflammatory signals through changes in ion channel activity, which are crucial for pain transmission. The (TRPV1) channel is key in mediating inflammatory pain, activated by thermal stimuli, low pH, and inflammatory mediators such as PGE2 and Bradykinin [29]. Activation of TRPV1 leads to calcium influx, membrane depolarization, and the initiation of pain signals, playing a critical role in hyperalgesia and allodynia. Another important group of channels, acid‐sensing ion channels (ASICs), especially ASIC3, are activated by acidic conditions in inflamed tissues, contributing to pain transmission [30]. Voltage‐gated sodium channels (Nav1.7, Nav1.8, and Nav1.9) are essential for action potential conduction in nociceptive neurons. These channels are regulated by inflammatory mediators, lowering the activation threshold and enhancing pain signaling, with dysregulation linked to chronic inflammatory and neuropathic pain [31].

Recent evidence has further expanded the understanding of ion channel involvement in nociception by highlighting the role of mechanosensitive ion channels, particularly Piezo1 and Piezo2. These channels are activated by mechanical stimuli such as pressure, stretch, and tissue deformation, thereby playing a central role in mechanotransduction within sensory neurons. Emerging findings indicate that Piezo channels contribute significantly to both inflammatory and mechanical hypersensitivity, where their upregulation or sensitization may enhance nociceptor excitability under pathological conditions. The inclusion of these channels complements the established roles of TRPV1, acid‐sensing ion channels (ASICs), and voltage‐gated sodium (Nav) channels, providing a more comprehensive and integrated view of the molecular mechanisms underlying pain signaling pathways [32].

#### 3.2.3 Central Sensitization and Pain Amplification

**Figure 4:**
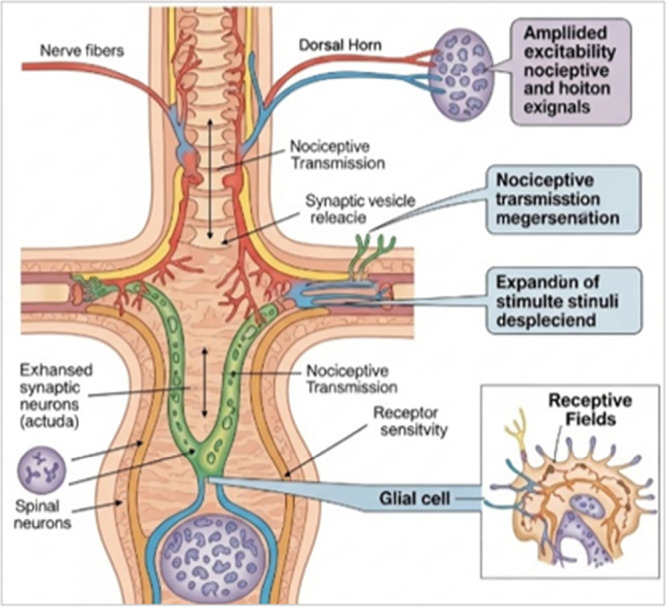
Central sensitization.

Central sensitization (Figure 4) refers to a maladaptive process in which the central nervous system (CNS) exhibits an exaggerated responsiveness to nociceptive input, resulting in heightened pain sensitivity even in the absence of significant peripheral stimulation. This phenomenon plays a pivotal role in the pathogenesis of chronic inflammatory pain [24]. At the spinal level, persistent nociceptive signaling amplifies synaptic transmission, primarily mediated by excitatory neurotransmitters such as glutamate and activation of *N*‐Methyl‐d‐Aspartate (NMDA) receptors, thereby augmenting neuronal excitability and facilitating long‐term potentiation of pain pathways [33].

Furthermore, glial cell activation, particularly microglia and astrocytes in response to tissue injury or inflammation leads to the release of pro‐inflammatory cytokines, including tumor necrosis factor‐alpha (TNF‐α), interleukin‐1 beta (IL‐1β), and interleukin‐6 (IL‐6). These mediators intensify neuronal excitability and sustain neuroinflammatory signaling, thereby perpetuating the sensitized state of central pain pathways. The neuroimmune crosstalk between neurons and glial cells thus represents a fundamental mechanism underlying the persistence and amplification of chronic inflammatory pain [34].

To strengthen the translational perspective, emerging evidence highlights those inflammatory mediators such as TNF‐α, IL‐1β, and IL‐6 are not only central to pain pathophysiology but also serve as potential clinical biomarkers for assessing disease activity and treatment response in inflammatory pain conditions [35]. In acute inflammatory pain, elevated levels of these biomarkers have been associated with increased pain intensity and may guide early therapeutic interventions [36].

Moreover, recent studies have identified Piezo1 and Piezo2 mechanosensitive ion channels as critical contributors to nociception. These channels are activated by mechanical stimuli and play a key role in converting physical forces into neuronal signals [37]. Dysregulation of Piezo channels has been implicated in various pain syndromes, including inflammatory and mechanical hypersensitivity states. Targeting these channels may represent a promising avenue for novel analgesic therapies. Collectively, integrating molecular biomarkers and emerging ion channel targets into clinical frameworks may enhance precision pain management and support more individualized, mechanism‐based therapeutic approaches [38].

This figure illustrates the mechanisms of central sensitization within the spinal cord, particularly at the dorsal horn. Persistent nociceptive input from peripheral tissues leads to enhanced synaptic transmission and increased excitability of spinal neurons. This process involves increased release of neurotransmitters, activation of postsynaptic receptors, and sensitization of nociceptive pathways [11]. As a result, there is amplification of pain signals, expansion of receptive fields, and heightened responsiveness to both painful and non‐painful stimuli. The figure also highlights the role of glial cell activation in maintaining and amplifying central sensitization, contributing to persistent and chronic pain states [18].

Recent advances in pain research have emphasized the clinical relevance of inflammatory biomarkers in the assessment and management of acute pain. Cytokines and other inflammatory mediators, including interleukin‐6 (IL‐6), tumor necrosis factor‐alpha (TNF‐α), and C‐reactive protein (CRP), have been shown to correlate with pain intensity and the degree of tissue injury. Emerging evidence suggests that these biomarkers may also serve as predictive indicators of treatment response, thereby supporting their potential role in guiding individualized therapeutic strategies. The integration of inflammatory biomarkers into pain assessment frameworks reinforces the concept of biomarker‐driven, precision pain management and enhances the translational value of mechanistic insights into clinical practice [39].

#### 3.2.4 Role of Neuropeptides in Inflammatory Pain

Neuropeptides, such as substance P and calcitonin gene‐related peptide (CGRP), are essential in modulating and propagating pain, especially during inflammation. Substance P, released from sensory neurons in response to harmful stimuli, binds to neurokinin‐1 (NK1) receptors on spinal cord neurons, facilitating pain transmission [30]. It also contributes to neurogenic inflammation by promoting the release of additional inflammatory mediators, amplifying the pain response [40]. Similarly, CGRP, which is co‐released with substance P during inflammation, enhances pain by increasing local blood flow, vascular permeability, and pro‐inflammatory cytokine release. Additionally, CGRP plays a significant role in central sensitization by altering the excitability of spinal dorsal horn neurons. These actions make CGRP a key target for developing new therapies for inflammatory and migraine‐related pain [41].

### 3.3 Blood–Brain Barrier Dysfunction and Immune System Interaction

The blood–brain barrier (BBB) serves as a crucial regulatory interface that preserves central nervous system (CNS) homeostasis by restricting the entry of immune cells and inflammatory mediators. However, under conditions of chronic inflammatory pain, the integrity of the BBB becomes compromised, thereby facilitating central sensitization and the persistence of pain [32]. During peripheral inflammation, pro‐inflammatory cytokines such as tumor necrosis factor‐alpha (TNF‐α), interleukin‐1 beta (IL‐1β), and interleukin‐6 (IL‐6) disrupt BBB function by modulating tight junction proteins and increasing vascular permeability [42]. Consequently, immune cells gain access to the CNS, where they interact with activated glial cells, notably microglia and astrocytes, to amplify neuroinflammatory signaling.

This neuroimmune interplay establishes a sustained pro‐inflammatory milieu within the CNS, which not only intensifies nociceptive transmission but also impairs inhibitory pain pathways. The resultant dysregulation reinforces central sensitization and may induce long‐lasting alterations in pain processing circuits, even after the resolution of the initial peripheral insult [43, 44].

### 3.4 Multidisciplinary Therapeutic Strategies of Inflammatory Pain

Inflammatory pain is a complex response to tissue damage triggered by pro‐inflammatory mediators that sensitize nerve endings and amplify pain [45]. Effective management often requires a multidisciplinary approach, targeting both the symptoms and root causes. Several therapeutic strategies aim to address these factors for comprehensive pain relief [46].

#### 3.4.1 Pharmacological Interventions

Nonsteroidal anti‐inflammatory drugs (NSAIDs) including ibuprofen, aspirin, and naproxen—alleviate inflammatory pain primarily through the inhibition of cyclooxygenase (COX) enzymes, leading to decreased prostaglandin synthesis and reduced inflammation. Although highly effective in managing pain associated with arthritis and musculoskeletal disorders, prolonged use is linked to adverse gastrointestinal, renal, and cardiovascular effects [47]. Corticosteroids (e.g., prednisone) exert potent anti‐inflammatory and immunosuppressive actions by downregulating inflammatory mediators, thereby providing therapeutic benefit in conditions such as rheumatoid arthritis and other autoimmune diseases. However, long‐term administration is associated with significant side effects, including osteoporosis, weight gain, and increased susceptibility to infection [48].

Disease‐modifying antirheumatic drugs (DMARDs), notably methotrexate and sulfasalazine target the immune system's pathogenic pathways, mitigating chronic inflammation and slowing disease progression in disorders like rheumatoid arthritis. Their use necessitates regular monitoring due to potential hepatotoxicity and bone marrow suppression [49]. Biologic agents, such as tumor necrosis factor (TNF) inhibitors and interleukin (IL) inhibitors, specifically neutralize key cytokines or immune cell targets, offering substantial efficacy in managing conditions such as rheumatoid arthritis and Crohn's disease. Despite their therapeutic benefits, these agents carry risks of infection and malignancy, warranting continuous clinical monitoring [50]. Topical analgesics, including NSAID formulations, capsaicin creams, and lidocaine patches, deliver localized analgesia for musculoskeletal and joint pain with minimal systemic exposure and fewer side effects [51].

#### 3.4.2 Physical Therapy and Rehabilitation

Exercise therapy improves joint mobility, reduces muscle stiffness, and enhances overall function by promoting endorphin release and improving blood circulation to inflamed tissues [52]. It is particularly beneficial for those with chronic conditions such as osteoarthritis and rheumatoid arthritis, with low‐impact exercises recommended to prevent worsening inflammation. Manual therapy, including joint mobilization and soft tissue manipulation, helps to alleviate pain and improve joint function, especially for those with arthritis and musculoskeletal pain [53, 54]. It should be performed by trained professionals to avoid injury. Thermotherapy (heat) relaxes muscles and boosts blood flow, while cryotherapy (cold) reduces swelling and numbs pain, which are both effective for managing acute inflammatory pain or muscle spasms, although care is needed to prevent skin damage [55].

#### 3.4.3 Psychological and Cognitive Strategies

Cognitive behavioral therapy (CBT) helps individuals to manage the emotional impact of chronic pain by changing negative thought patterns and teaching coping strategies, particularly for those with chronic inflammatory conditions [56]. It improves quality of life by addressing anxiety and depression. Mindfulness and relaxation techniques, such as meditation and progressive muscle relaxation, reduce pain perception by promoting relaxation and stress relief, with mindfulness‐based stress reduction (MBSR) being commonly used for pain management [57]. Biofeedback uses sensors to monitor physiological responses and provides real‐time feedback to help control them, reducing pain and stress, especially in chronic inflammatory pain. However, it requires training and may take time for noticeable benefits [58].

#### 3.4.4 Interventional, Nutritional, and Supplementary Approaches

Corticosteroid and local anesthetic injections offer short‐term relief by attenuating inflammation and pain in conditions such as arthritis and tendonitis. While effective, repeated administration may result in tissue degeneration or joint weakening, necessitating cautious use under medical supervision [59]. Nerve blocks, which involve the targeted injection of local anesthetics to interrupt nociceptive transmission along specific neural pathways, provide substantial relief for severe or refractory inflammatory pain, though their application requires specialized clinical oversight [60]. Radiofrequency ablation (RFA) represents a more invasive intervention, utilizing thermal energy from radio waves to disrupt nociceptive nerve conduction. It can produce long‐lasting analgesia in patients with chronic pain syndromes unresponsive to conventional therapies, though procedural risks must be carefully weighed [61].

In parallel, nutritional interventions play a supportive role in modulating inflammation. An anti‐inflammatory diet enriched with antioxidants, omega‐3 fatty acids, and bioactive compounds such as turmeric and ginger have been shown to help regulate inflammatory pathways and reduce pain perception [62]. This dietary strategy is frequently employed adjunctively with standard pharmacological treatments in conditions like rheumatoid arthritis and inflammatory bowel disease [63]. Moreover, nutraceutical supplements including omega‐3 fatty acids, glucosamine, chondroitin, and curcumin have demonstrated potential in attenuating inflammation and alleviating musculoskeletal discomfort. However, their use should be individually assessed and medically supervised to ensure both efficacy and safety [64].

#### 3.4.5 Alternative Therapies

Several non‐pharmacological therapies support chronic inflammatory pain management. Acupuncture stimulates the nervous system through needle insertion and is commonly used for conditions such as arthritis [65]. TENS delivers low‐level electrical currents for pain relief, though tolerance may develop with frequent use. Chiropractic care involves spinal adjustments, which are especially effective for musculoskeletal and inflammatory arthritis pain when integrated with other treatments [66]. Light therapy (e.g., infrared, laser) reduces pain and promotes healing via anti‐inflammatory and neural pathways. Ultrasound therapy uses sound waves to reduce inflammation, promote tissue repair, and modulate pain signals [67]. Hypnosis is a psychological therapy that uses guided suggestions to alter consciousness, helping patients to manage chronic pain by changing how it is perceived in sensory, emotional, and cognitive ways [68].

It also helps to reduce negative emotions such as fear and anxiety while promoting relaxation and improved coping strategies. Mindfulness‐based interventions focus on cultivating non‐judgmental awareness to help individuals respond to pain more adaptively [69]. Placebo and nocebo effects describe how expectations and beliefs about treatment can influence pain perception, with positive expectations reducing pain (placebo) and negative beliefs worsening it (nocebo). Optimizing placebo effects and minimizing nocebo responses can enhance pain management outcomes [70]. Yoga and Tai Chi are mind/body practices that help to alleviate chronic pain and improve well‐being through physical postures, movements, breathing, and mental focus. They support pain management by regulating bodily functions [71]. Several complementary therapies support chronic pain management by targeting the nervous system. Music therapy uses interventions such as listening and improvisation to reduce pain perception. Manual and movement therapies apply hands‐on techniques and exercise to restore function and modulate pain through neural mechanisms. Biofeedback trains individuals to control physiological responses, aiding in pain regulation [55]. Virtual reality (VR) therapy provides immersive environments that distract and stimulate the brain, promoting neuroplasticity and altering the processing of pain [72].

Understanding the molecular mechanisms underlying inflammatory pain provides a critical foundation for developing effective, patient‐centered care strategies. This biochemical knowledge not only informs pharmacological approaches but also enhances the clinical reasoning of healthcare professionals, particularly nurses [14, 15, 16]. By recognizing how inflammatory mediators influence pain pathways, nurses can better assess symptom patterns, anticipate treatment responses, and tailor non‐pharmacological interventions. Integrating molecular insights into nursing practice supports more precise, evidence‐based care, aligning with the principles of precision health and strengthening the nurse's role within multidisciplinary pain management frameworks [45, 46, 47, 48, 49, 50, 51, 52, 53, 54, 55, 56, 57].

### 3.5 The Nursing Role in Addressing Inflammatory Pain: Precision and Presence

Inflammatory pain, commonly associated with conditions such as arthritis, autoimmune disorders, and infections, poses a significant challenge in the healthcare context. This type of pain can severely affect a patient's physical, emotional, and psychological health [73]. Nurses are crucial in managing inflammatory pain, serving as a bridge between medical treatments and patient‐focused care. Through emphasizing both precision (through thorough pain assessment and personalized treatment) and presence (by offering compassionate support and guidance), nurses play a vital role in improving patient experiences and pain management outcomes [42]. This multifaceted responsibility involves nurses thoroughly assessing pain, implementing suitable interventions, educating patients on managing their conditions, and advocating for continuous care and assistance [74].

By combining clinical knowledge, empathy, and consistent monitoring, nurses ensure that inflammatory pain is effectively treated while empowering patients to take charge of their own comfort [13]. Thus, the nursing profession uniquely merges scientific expertise with compassionate care, applying both technical proficiency and emotional sensitivity to the management of pain. Nurses hold a critical position in the comprehensive management of inflammatory pain, playing a dynamic role in assessment, intervention, education, and patient advocacy. Their approach blends clinical knowledge, individualized care, and interdisciplinary teamwork, which are all vital for effectively addressing the complex nature of inflammatory pain [7, 8, 9, 10, 11, 12, 13, 14, 15, 16, 17, 18, 19].

### 3.6 Holistic Evaluation and Early Detection

Nurses are well equipped to carry out ongoing, in‐depth evaluations that encompass not only physical symptoms, but also emotional, psychological, and social factors related to pain [75]. Utilizing standardized pain assessment tools, they can detect the onset and progression of inflammatory pain, recognize patterns, and gauge the effectiveness of interventions. Additionally, they monitor related symptoms such as fatigue, low mood, and frequent reduced mobility challenges in chronic inflammatory conditions such as lupus and rheumatoid arthritis [49, 50, 51, 52, 53, 54, 55, 56, 57, 58, 59, 60, 61, 62, 63].

### 3.7 Educating and Empowering Patients

A key component of nursing care involves patient education. Nurses explain the biological basis of inflammation and pain, the significance of the following prescribed medications (e.g., NSAIDs, DMARDs, and biologics), and the positive impact of lifestyle changes, including healthy eating, physical activity, and quitting smoking [1]. They also introduce patients to complementary approaches such as heat therapy or mindfulness techniques. This educational role fosters patient autonomy and supports long‐term self‐management [76].

### 3.8 Coordinating Multimodal Treatment Approaches

Nurses implement diverse pain management plans that are tailored to each patient's needs. Working collaboratively with doctors, physiotherapists, psychologists, and other healthcare providers, they help to develop and execute comprehensive treatment strategies [13]. These often involve medication management, facilitation of physical therapies, psychological interventions, and alternative techniques such as massage or guided imagery. Nurses continually evaluate outcomes and adjust care to enhance comfort and reduce adverse effects [77].

### 3.9 Emotional Support and Therapeutic Presence

Chronic inflammatory pain often has significant emotional ramifications. Nurses provide essential psychosocial support through empathetic communication, encouragement, and therapeutic presence [78]. They help patients to manage psychological distress such as anxiety and depression by establishing trust and creating a safe environment. Simply being present and attentive can foster healing and ease the emotional burden of chronic pain [10, 11, 12, 13, 14, 15, 16, 17, 18, 19, 20, 21, 22, 23, 24, 25, 26, 27, 28, 29, 30, 31, 32, 33, 34, 35, 36, 37, 38, 39, 40, 41, 42, 43, 44, 45, 46, 47, 48, 49, 50, 51, 52, 53, 54, 55, 56, 57, 58, 59, 60, 61, 62, 63, 64, 65, 66, 67, 68, 69, 70, 71, 72, 73, 74, 75, 76, 77, 78, 79].

### 3.10 Personalized and Data‐Driven Care Planning

As precision healthcare continues to evolve, nurses are increasingly adopting individualized approaches that incorporate genetic profiles, biomarkers, and psychosocial factors [54]. This allows them to create more personalized care plans that address both the physiological and emotional aspects of pain. By aligning interventions with each patient's unique responses, nurses can ensure more effective and targeted pain management [80]. For example, understanding a patient's genetic makeup can guide medication choices, reducing the risk of adverse reactions or drug interactions. Additionally, considering psychosocial elements such as stress or mental health conditions enables nurses to provide a more holistic approach to pain management [19]. This personalized strategy not only improves treatment accuracy but also enhances patient outcomes and overall quality of life for those dealing with inflammatory pain. By integrating clinical expertise with data‐driven insights, nurses can offer care that is both efficient and tailored to the individual needs of each patient [66].

### 3.11 Ethical Practice and Advocacy

As advocates, nurses safeguard equitable access to pain management resources while ensuring that the care provided remains ethical and patient‐centered. Nurses play a vital role as advocates in ensuring equitable access to pain management resources while maintaining ethical, patient‐centered care [81]. They help patients to understand their treatment options, both pharmacological and non‐pharmacological, fostering informed decision‐making and active participation in care. Nurses face the challenge of balancing effective pain relief with the risk of medication misuse or dependency, working to prevent over‐prescription, educating patients on safe usage, and promoting alternative therapies such as physical therapy and cognitive behavioral therapy [59]. By respecting patients' autonomy and guiding them through ethical dilemmas, nurses empower patients to manage their pain effectively. Through advocacy, education, and ethical decision‐making, nurses provide compassionate care that addresses both physical and emotional well‐being [79].

### 3.12 Advancing Practice Through Research

Nurses play an indispensable role in advancing the science and practice of inflammatory pain management through their active engagement in research, clinical audits, and the formulation of evidence‐based guidelines. Participation in research enables nurses to contribute to the expanding body of knowledge surrounding pain pathophysiology, therapeutic efficacy, and patient‐centered outcomes [21]. Their unique clinical proximity to patients provides a valuable perspective for observing the real‐world impact of diverse pain management strategies, thereby generating contextual insights that can refine and optimize treatment protocols.

Through clinical audits, nurses systematically evaluate the effectiveness and quality of existing pain management interventions, identifying gaps and ensuring that clinical practices remain aligned with current evidence and professional standards [79]. Furthermore, their involvement in developing and updating best practice guidelines allows nurses to influence institutional policies and standardize multidisciplinary approaches to inflammatory pain management across healthcare settings. By integrating clinical expertise with scientific inquiry, nurses not only enhance the translation of research into practice but also ensure that pain management frameworks remain responsive to patient experiences and emerging evidence. Their continuous feedback loop between bedside care and evidence‐based innovation positions nursing professionals as central agents in the evolution of precision, compassionate, and effective inflammatory pain care [22, 24, 25, 26, 27, 28, 29, 30, 31, 32, 33, 34, 35, 36, 37, 38, 39, 40, 41, 42, 43, 44, 45, 46, 47, 48, 49, 50, 51, 52, 53, 54, 55, 56, 57, 58, 59, 60, 61, 62, 63, 64, 65, 66, 67, 68, 69, 70, 71, 72, 73, 74, 75, 76, 77, 78, 79, 80, 81].

## 4 Conclusions

Inflammatory pain represents a multifactorial condition orchestrated by intricate molecular mechanisms, including nociceptor sensitization, central sensitization, and the release of pro‐inflammatory mediators. Its effective management necessitates a multidisciplinary framework that integrates pharmacological therapies (e.g., NSAIDs, corticosteroids, biologics), physical rehabilitation techniques (e.g., exercise and manual therapy), and psychological interventions such as CBT and mindfulness‐based strategies. In addition, emerging modalities—including virtual reality, acupuncture, and photo‐biomodulation (light therapy)—demonstrate growing potential as adjuncts to conventional treatments. Within this integrative context, nurses play a pivotal role by providing precise, evidence‐informed, and empathetic care, bridging molecular understanding with clinical application to optimize patient outcomes.

Future research should aim to translate molecular discoveries into clinical nursing practice through the identification and validation of biomarkers for early detection and monitoring of inflammatory pain, and through the integration of genetic and epigenetic profiling to enable personalized nursing interventions. Moreover, embedding molecular and precision care concepts within nursing education and training will strengthen the foundation for tailored, science‐driven practice. The incorporation of digital health technologies, such as wearable biosensors and mobile applications, alongside nursing supervision, offers opportunities for real‐time pain assessment and management. Equally important is the exploration of culturally sensitive, patient‐centered strategies and the evaluation of the long‐term effectiveness of innovative therapies within nursing‐led multidisciplinary programs. Collectively, these advancements are expected to bridge the gap between molecular science and clinical nursing, fostering a new era of personalized, integrative, and effective care for individuals experiencing inflammatory pain.

## 5 Relevance and Clinical Implications

Bridging molecular mechanisms with clinical practice is essential for advancing the management of inflammatory pain. Key inflammatory mediators, including tumor necrosis factor‐alpha (TNF‐α), interleukin‐1 beta (IL‐1β), and interleukin‐6 (IL‐6), are not only central to the pathophysiology of pain but also hold potential as clinically relevant biomarkers for assessing disease activity, pain severity, and response to treatment. Elevated levels of these cytokines have been associated with heightened nociceptor sensitization and increased pain perception, supporting their use in guiding targeted anti‐inflammatory and analgesic therapies. Moreover, the identification of molecular pathways such as NF‐κB and MAPK has facilitated the development of pharmacological interventions aimed at modulating inflammatory signaling cascades.

At the neurophysiological level, alterations in ion channel activity, including TRPV1, Nav1.7, and Nav1.8, translate into clinically observable phenomena such as hyperalgesia and allodynia. Emerging evidence on mechanosensitive ion channels, particularly Piezo1 and Piezo2, further expands the understanding of pain mechano‐transduction and offers promising targets for novel therapeutic strategies. Importantly, integrating these molecular insights into clinical decision‐making supports a precision medicine approach, enabling more individualized pain assessment and management. Within this framework, nurses play a pivotal role in translating evidence into practice through comprehensive pain assessment, monitoring biomarker‐informed interventions, and delivering holistic, patient‐centered care.

## Author Contributions

**Fatma Mohamed Elmansy:** conceptualization, validation, project administration, supervision. **Mohamed Goda Elbqry:** data curation, project administration, visualization, writing – original draft, investigation, writing – review and editing. **Saddam Ahmed Al‐Ahdal:** investigation, validation, formal analysis, supervision, visualization. **Fatima S. O. Ashmieg:** methodology, conceptualization, software, data curation, resources, project administration, investigation. **Patience Osose Nasir:** conceptualization, funding acquisition, visualization, formal analysis, supervision, resources, project administration. **Nasiru Mohammed Abdullahi:** methodology, conceptualization, visualization, formal analysis. **Mohammed Nader Shalaby:** conceptualization, investigation, funding acquisition, writing – original draft, writing – review and editing, visualization, validation, methodology, software, formal analysis, project administration, resources, supervision, data curation.

## Ethics Statement

This study is a narrative review based on previously published literature and does not involve human participants, patient data, or animal subjects. Therefore, ethical approval and informed consent were not required. All sources were appropriately cited, and the review was conducted in accordance with accepted ethical standards for research and publication.

## Consent

The authors have nothing to report.

## Conflicts of Interest

The authors declare no conflicts of interest.

## Transparency Statement

The corresponding author, Mohammed Nader Shalaby, affirms that this manuscript is an honest, accurate, and transparent account of the study being reported; that no important aspects of the study have been omitted; and that any discrepancies from the study as planned (and, if relevant, registered) have been explained.

## Data Availability

The authors have nothing to report.
